# The plant cysteine oxidases from *Arabidopsis thaliana* are kinetically tailored to act as oxygen sensors

**DOI:** 10.1074/jbc.RA118.003496

**Published:** 2018-05-30

**Authors:** Mark D. White, Jos J. A. G. Kamps, Samuel East, Leah J. Taylor Kearney, Emily Flashman

**Affiliations:** From the Chemistry Research Laboratory, University of Oxford, 12 Mansfield Road, Oxford OX1 3TA, United Kingdom

**Keywords:** hypoxia, enzyme kinetics, protein degradation, Arabidopsis, plant biochemistry, post-translational modification (PTM), ERF-VII, N-end rule, oxygen-sensing, plant cysteine oxidase

## Abstract

Group VII ethylene response factors (ERF-VIIs) regulate transcriptional adaptation to flooding-induced hypoxia in plants. ERF-VII stability is controlled in an O_2_-dependent manner by the Cys/Arg branch of the N-end rule pathway whereby oxidation of a conserved N-terminal cysteine residue initiates target degradation. This oxidation is catalyzed by plant cysteine oxidases (PCOs), which use O_2_ as cosubstrate to generate Cys-sulfinic acid. The PCOs directly link O_2_ availability to ERF-VII stability and anaerobic adaptation, leading to the suggestion that they act as plant O_2_ sensors. However, their ability to respond to fluctuations in O_2_ concentration has not been established. Here, we investigated the steady-state kinetics of *Arabidopsis thaliana* PCOs 1–5 to ascertain whether their activities are sensitive to O_2_ levels. We found that the most catalytically competent isoform is AtPCO4, both in terms of responding to O_2_ and oxidizing AtRAP2.2/2,12 (two of the most prominent ERF-VIIs responsible for promoting the hypoxic response), which suggests that AtPCO4 plays a central role in ERF-VII regulation. Furthermore, we found that AtPCO activity is susceptible to decreases in pH and that the hypoxia-inducible AtPCOs 1/2 and the noninducible AtPCOs 4/5 have discrete AtERF-VII substrate preferences. Pertinently, the AtPCOs had *K*_*m*(O2)_^app^ values in a physiologically relevant range, which should enable them to sensitively react to changes in O_2_ availability. This work validates an O_2_-sensing role for the PCOs and suggests that differences in expression pattern, ERF-VII selectivity, and catalytic capability may enable the different isoforms to have distinct biological functions. Individual PCOs could therefore be targeted to manipulate ERF-VII levels and improve stress tolerance in plants.

## Introduction

Plants are sessile organisms that require sufficient mechanisms to effectively respond to low-oxygen conditions (hypoxia), such as those experienced during submergence or waterlogging, to survive. Molecular adaptation to hypoxia is mediated by group VII ethylene response factors (ERF-VIIs),^5^ transcriptional activators that up-regulate the expression of core anaerobic genes during periods of low oxygen (1, 2). At a physiological level, this can result in an escape strategy whereby petioles/shoots elongate above the surface to improve O_2_ supply (mediated by the ERF-VIIs SNORKEL 1 and SNORKEL 2 in deep-water rice varieties (3)) or in a quiescence response whereby plants enter a temporary anaerobic state to conserve O_2_ reserves until floodwaters subside (mediated by the ERF-VIIs HRE1/2 and RAP2.2 in *Arabidopsis* (4, 5)).

Five members of the ERF-VII family have been identified in *Arabidopsis thaliana* (RAP2.2, RAP2.3, RAP2.12, HRE1, and HRE2), all of which have the conserved N-terminal motif CGGA(I/V)ISD(F/Y) following cotranslational methionine excision. ERF-VII stability is controlled in an O_2_-dependent manner by the Cys/Arg branch of the N-end rule pathway (6, 7). During hypoxia, the ERF-VIIs can translocate to the nucleus to promote the expression of anaerobic genes. Under normoxic conditions, their N-terminal cysteine residues are oxidized, promoting subsequent arginylation (8), ubiquitination (9), and degradation (2, 10) of the target by arginyltransferases, the E3 ligase proteolysis 6, and the 26S proteasome, respectively. Furthermore, as well as regulating the plant hypoxic response, N-end rule–mediated ERF-VII stability has been linked to other abiotic stress responses (2, 11, 12).

ERF-VII N-terminal cysteine oxidation has been shown to be enzymatically regulated *in vitro* and in *Arabidopsis* by the plant cysteine oxidases (8, 13). These monomeric, nonheme iron (Fe^2+^)-dependent dioxygenases use both atoms of molecular O_2_ to promote the formation of Cys-sulfinic acid, which is a suitable and sufficient substrate for arginyltransferase 1–catalyzed arginylation, at the N terminus of their ERF-VII targets (8). The direct use of molecular O_2_ to promote the proteasomal degradation of primary anaerobic response elements has led to the suggestion that the PCOs function as plant O_2_ sensors (13). In animals, the hypoxic response is coordinated by hypoxia-inducible transcription factor (HIF) levels, which are regulated by Fe^2+^, 2-oxoglutarate, and O_2_-depedendent HIF prolyl hydroxylase enzymes (PHDs) in an analogous manner to the ERF-VIIs and PCOs (14). Hydroxylation of specific prolyl residues in HIF-α (coupled with 2-oxoglutarate decarboxylation to succinate and CO_2_) targets HIF-α for proteasomal degradation (14). These enzymes are termed O_2_ sensors due to the dependence of their rate of activity on O_2_ availability. This is biochemically defined by high *K*_*m*(O2)_^app^ values, particularly for the primary O_2_ sensor, PHD2 (240–1700 μm (15–18)). For the PCOs to effectively function in the same capacity, their rate of activity must also be sensitive to O_2_ availability in a physiologically relevant manner.

In this study, we sought to determine whether the PCOs have the biochemical potential to act as O_2_ sensors via a steady-state kinetic investigation. We have optimized assay conditions for analyzing PCO activity and determined the kinetic parameters of each *A. thaliana* PCO isoform (AtPCO) in terms of pH, a peptide representing the N termini of the AtERF-VIIs AtRAP2.2/2.12, and O_2_. Importantly, we report that the AtPCOs have *K*_*m*(O2)_^app^ values that fall within a physiologically relevant range, implicating their biochemical capacity to act as O_2_ sensors in plants. We also found that each isoform possesses distinct catalytic capability and substrate specificity, which may correlate with expression behavior. Consequently, our work provides biochemical evidence supporting the assertion that the AtPCOs act as plant O_2_ sensors and that suggests that different isoforms could have divergent biological functions.

## Results

### Recombinant production of AtPCOs 1–5

**Figure 1.**
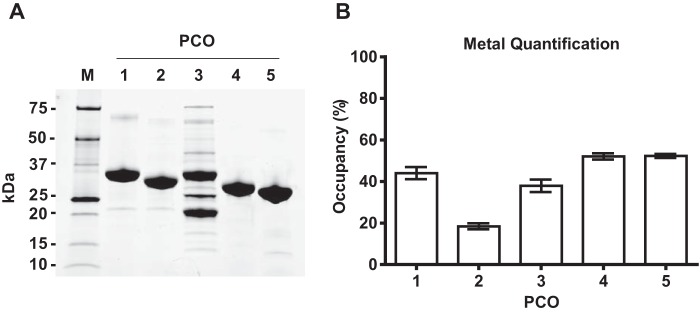
**Purification and iron content of AtPCOs 1–5.** *A*, SDS-PAGE analysis showing purified AtPCO isoforms 1–5 following sequential steps of immobilized nickel-affinity and size-exclusion chromatography with *M* indicating the molecular mass marker. Each AtPCO protein migrated to the expected mass, demonstrating over 95% purity, except for AtPCO3, which was estimated to be ∼50% pure. This was accounted for in all activity measurements. *B*, the iron content of each AtPCO was quantified using a ferrozine-like assay, allowing metal occupancy to be estimated from known protein concentrations. All samples possessed substoichiometric amounts of cofactor with AtPCO2 containing the least iron. *Error bars* display S.D. (*n* = 3).

We have previously reported the recombinant production of *A. thaliana* AtPCOs 1 and 4 to over 90% purity by immobilized nickel-affinity chromatography (8). In the current study, all five AtPCO isoforms were subcloned into the expression vector pET28a and purified with an additional size-exclusion chromatography step to improve protein quality. Each AtPCO was obtained at over 95% purity (Fig. 1*A*) except for AtPCO3, which appeared to be ∼50% pure by SDS-PAGE despite generating a size-exclusion elution profile similar to the other isoforms. Proteomic analysis suggests that the additional bands correspond to degraded fragments of AtPCO3 and contaminating *Escherichia coli* proteins (data not shown). Consistent with previous findings (8), iron quantification using a ferrozine-like assay revealed that each AtPCO isoform binds substoichiometric amounts of Fe^2+^ (∼20–50%; Fig. 1*B*) with AtPCO2 possessing the lowest measure of metal.

### AtPCOs 1–5 catalyze oxidation of AtRAP2(2–15)

AtPCOs 1 and 4 can catalyze the dioxygenation of cysteines situated at the N terminus of 10-mer peptides representing AtERF-VII substrates (8). For this study, we wished to confirm that all five AtPCO isoforms can catalyze the oxidation of cysteines positioned at the N terminus of a 14-amino acid–long peptide corresponding to the methionine excised N terminus of AtRAP2.2 and AtRAP2.12 (CGGAIISDFIPPPR), hereby referred to as AtRAP2(2–15) (14-mer peptides allowed distinction between AtRAP2(2–15) and other AtERF-VII peptides; see below). AtRAP2.2 and AtRAP2.12 have been reported as two of the most prominent activators of anaerobic genes in *Arabidopsis*; therefore, understanding their regulation will provide valuable information on molecular adaptation to hypoxia in plants (5, 19, 20).

AtRAP2.2(2–15) was incubated with AtPCOs 1–5 at 25 °C for 30 min before being quenched with 1% formic acid and analyzed by liquid chromatography–coupled mass spectrometry (LC-MS). As expected, AtRAP2(2–15) was a suitable substrate for all AtPCO isoforms with complete conversion observed in each sample (Fig. S1).

**Figure 2.**
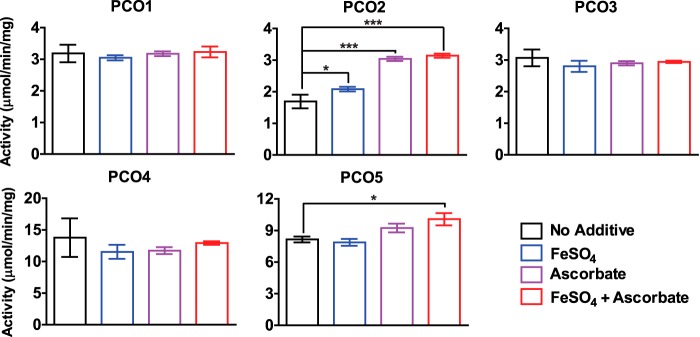
**Analyzing the influence of exogenous iron and ascorbate on AtPCO activity.** The specific activities of AtPCOs 1–5 were determined with and without FeSO_4_ (100-fold in excess of enzyme concentration) and sodium ascorbate (1 mm) by measuring the rate of AtRAP2(2–15) cysteine oxidation at regular time intervals (0, 30, 60, and 90 s) by LC-MS. The addition of exogenous additives, particularly ascorbate, significantly increased the specific activities of AtPCO2 and, to a lesser extent, AtPCO5 but had little effect on the other isoforms. AtPCOs 4 and 5 had the greatest specific activity. Reactions were conducted in 50 mm HEPES, 50 mm NaCl, and 1 mm TCEP, pH 7.5, at 25 °C. Statistical analysis was completed using a one-way analysis of variance, post hoc Dunnett test using the “no additive” sample as the control reference with * and *** denoting *p* ≤ 0.05 and *p* ≤ 0.001, respectively. *Error bars* display S.D. (*n* = 3).

We subsequently determined whether the addition of exogenous Fe^2+^ and/or ascorbate would enhance the activity of the AtPCOs, analogous to other nonheme iron–dependent oxygenases (21, 22), by comparing the specific activities of AtPCOs 1–5 with and without FeSO_4_, ascorbate, or both. Stopped assays were conducted at 0-, 30-, 60-, and 90-s intervals to monitor the initial rate of AtRAP2(2–15) oxidation by LC-MS. The addition of both Fe^2+^ and ascorbate significantly increased the specific activity of AtPCO2 and to a lesser extent AtPCO5 but had little or no effect on the activity of the other isoforms (Fig. 2). In the case of AtPCO2, this could be attributed to enhanced iron occupancy, compensating for the low quantities estimated in Fig. 1*B*. In light of these results, both additives were retained in all subsequent reactions to facilitate optimal AtPCO activity, and turnover rates are reported per mg of total enzyme present. It is important to note that the specific activities of each isoform varied significantly under equivalent conditions with AtPCOs 4 and 5 catalyzing AtRAP2(2–15) oxidation at ∼13 and 10 μmol/min/mg, respectively, compared with AtPCOs 1, 2, and 3, which processed AtRAP2(2–15) at around 3 μmol/min/mg (taking into account the 50% purity of AtPCO3).

### The activities of AtPCOs 1–5 are pH-dependent

The specific activities of AtPCOs 1–5 were tested between pH 6.0 and 9.0 to determine the optimum buffering conditions for onward kinetic investigation. Bis tris propane was selected for analysis because of its broad buffering capacity, preventing any variability in activity caused by different buffer molecules. Initial rates of AtPCO activity were measured at 0.5 pH intervals using the LC-MS–based assay described above.

**Figure 3.**
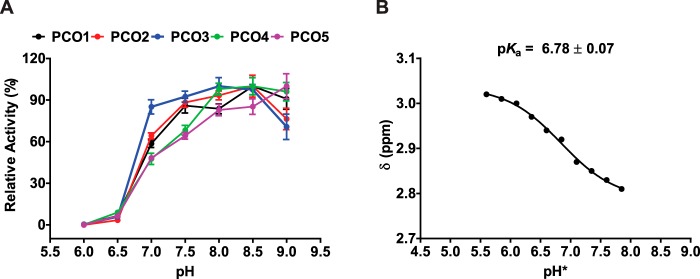
**The activity of AtPCOs 1–5 depends on pH.** *A*, pH profile showing the relative activities of AtPCOs 1–5 with 200 μm RAP2(2–15) at 25 °C and different pH values using 50 mm Bis tris propane, 50 mm NaCl, and 5 mm TCEP as buffer. A significant reduction in AtPCO activity is observed below pH 7.0 for all isoforms. This correlates with the side-group p*K_a_* for the N-terminal cysteine of RAP2(2–15) (*B*), which was determined by monitoring the chemical shift of cysteine β-protons in D_2_O (>95%) using ^1^H NMR. This suggests that the thiol needs to be deprotonated for binding and/or turnover. pH* denotes the pH reading given by a pH meter calibrated for solutions in H_2_O for D_2_O samples. p*K_a_* was corrected to H_2_O using the formula *pK*^H^ = 0.929*pK*^H^* + 0.42 derived in Ref. 41. *Error bars* display S.E. (*n* = 3).

All isoforms were observed to exhibit a similar pH profile, showing preference for slightly basic conditions (Fig. 3*A*). Maximum activity was achieved at pH 8.0 for AtPCO3; pH 8.5 for AtPCOs 1, 2, and 4; and pH 9.0 for AtPCO5. Interestingly, a significant reduction in AtPCO activity was seen for all AtPCO isoforms below pH 7.0 with almost all enzyme activity eliminated at pH 6.0. ^1^H NMR analysis of the AtRAP2(2–15) peptide revealed that the thiol group p*K_a_* of the N-terminal cysteine is 6.78 (Fig. 3*B* and Fig. S2), at least 1.0 pH unit lower than the free amino acid, suggesting that the thiol is deprotonated for binding and/or turnover.

Collectively, the highest level of activity for all AtPCO isoforms was observed at pH 8.0 and 8.5. The former was selected for all subsequent kinetic analysis to combine optimal activity with the most physiologically relevant conditions.

### AtPCOs 1–5 have different catalytic capacities toward AtRAP2(2–15) under aerobic conditions

The kinetic parameters of AtPCOs 1–5 for AtRAP2(2–15) were determined under atmospheric conditions as a prelude to establishing the O_2_ sensitivities of each isoform. As well as identifying the peptide concentrations needed to generate saturating conditions for *K_m_*_(O2)_ determination, a comparison of kinetic values for AtRAP2.2 and -2.12, two of the most prominent activators of anaerobic adaptation (19, 20), may contribute to a greater understanding of the physiological role of each AtPCO isoform during the plant hypoxic response. Initial rates of AtPCO activity (Fig. S3) were determined at varying AtRAP2(2–15) concentrations using the assay described above before fitting to the Michaelis–Menten model to estimate catalytic parameters.

**Figure 4.**
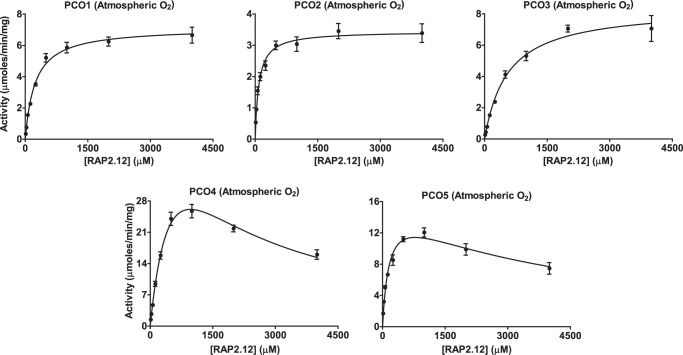
**Analyzing the dependence of AtPCOs 1–5 activity on AtRAP2(2–15) availability under atmospheric O_2_.** Michaelis–Menten kinetic plots for AtPCOs 1–5 with respect to AtRAP2(2–15) concentration (calculated from initial rates presented in Fig. S3) are shown. Assays were conducted under aerobic conditions at 25 °C using 50 mm Bis tris propane, 50 mm NaCl, and 5 mm TCEP, pH 8.0, as buffer. Data collected for AtPCO4 and AtPCO5 were fitted to an equation for substrate inhibition to address the decline in rate at high RAP2(2–15) concentrations. *Error bars* display S.E. (*n* = 3).

The Michaelis–Menten plots (Fig. 4) and derived kinetic constants (Table 1) indicate striking differences between each isoform. AtPCO4 had the highest turnover number at 31.0 s^−1^, whereas AtPCOs 1, 3, and 5 had turnover values of 4.18, 4.70, and 7.90 s^−1^, respectively. AtPCO2 was the least active with a *k*_cat_ of 1.98^−1^. AtPCOs 4 and 5 exhibited a decline in rate at AtRAP2(2–15) concentrations above ∼1 mm, which could be fitted to a standard equation for substrate inhibition, resulting in an inhibition constant of 1.4 mm for AtPCO4 and 3.8 mm for AtPCO5. *K_m_* values for AtRAP2(2–15) ranged from ∼88 μm for AtPCO2 to 690 μm for AtPCO4 with AtPCOs 1, 3, and 5 generating Michaelis constants of 238, 560, and 161 μm, respectively.

**Table 1:** **Steady-state kinetic parameters of AtPCOs 1–5 toward AtRAP2(2–15) at atmospheric O_2_** The turnover numbers (*k*_cat_), Michaelis constants (*K_m_*), and related maximum velocities (*V*_max_) of AtPCOs 1–5 for RAP2(2–15) were calculated by analyzing the initial rate of AtPCO-catalyzed cysteine oxidation (Fig. S3) as a subject of AtRAP2(2–15) concentration using the Michaelis–Menten model of enzyme kinetics (Fig. 4). Substrate inhibition was observed for AtPCOs 4 and 5 with inhibition constants calculated as 1.35 ± 0.24 and 3.78 ± 1.03 mm, respectively.

AtPCO	*k*_cat_	*K_m_*	*V*_max_
	*s*^−*1*^	*mm*	μ*mol min*^−*1*^ *mg*^−*1*^
1	4.18 ± 0.09	0.24 ± 0.02	7.14 ± 0.16
2	1.98 ± 0.04	0.09 ± 0.01	3.45 ± 0.08
3	4.70 ± 0.17	0.56 ± 0.06	8.43 ± 0.31
4	31.0 ± 3.49	0.69 ± 0.11	63.4 ± 7.13
5	7.90 ± 0.74	0.16 ± 0.03	16.1 ± 1.51

Overall the kinetic data strongly indicated that AtPCO4 is the most catalytically potent isoform despite possessing a relatively high *K_m_* for AtRAP2(2–15) as the large turnover number of AtPCO4 overcomes the weak substrate association. Furthermore, the substrate inhibition exhibited by AtPCO4 is only observed at high AtRAP2(2–15) concentrations, which may not be biologically relevant.

### The K_m(O2)_^app^ values for AtPCOs 1–5 are consistent with an O_2_-sensing role

For enzymes to act as O_2_ sensors, their activity must be rate-limited by O_2_ availability in a physiologically relevant range. This can be predicted by comparing biochemically determined *K_m_*_(O2)_ values with reported (sub)cellular O_2_ concentrations when available. To ascertain whether the AtPCOs have the biochemical potential to act as O_2_ sensors, we determined the *K*_*m*_^app^ of each isoform for O_2_ using steady-state kinetic assays.

To ensure that AtPCO dependence on O_2_ was not limited by AtRAP2(2–15) availability, peptide concentrations of 5 times the respective *K_m_*_(AtRAP2(2–15))_ value were used for analysis (Table 1). A higher AtRAP2(2–15) concentration (for example 10 times the *K_m_*) was not selected both to conserve substrate stocks and to avoid substrate inhibition of AtPCO5 (Fig. 4). Unfortunately, this criterion could not avoid the more significant substrate inhibition region of AtPCO4; therefore, the O_2_ dependence of this isoform was tested using a concentration of peptide that provided a maximum rate of AtPCO4 activity under aerobic conditions (1 mm).

Steady-state kinetic assays were conducted at different O_2_ concentrations using a method established previously in our laboratory (17). AtRAP2(2–15) solutions were prepared in gas-tight MS vials and equilibrated with different ratios of O_2_ and N_2_ before adding enzyme to initiate the reaction. Single time points within the linear rate ranges established above (Fig. S3) were taken by quenching the reaction with formic acid, allowing AtRAP2(2–15) oxidation to be quantified by LC-MS analysis.

**Figure 5.**
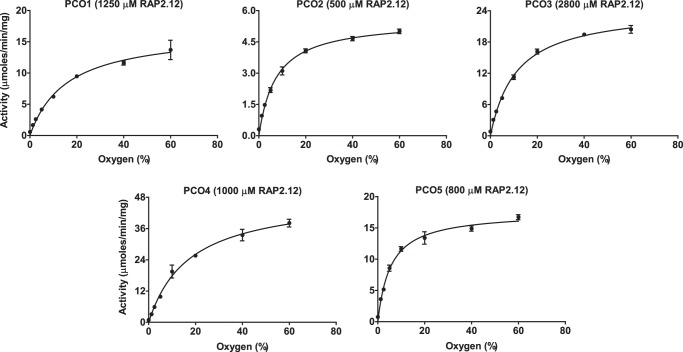
**Dependence of AtPCOs 1–5 activity on O_2_ availability.** Michaelis–Menten kinetic plots display the activities of AtPCOs 1–5 with respect to O_2_ concentration (using single time points within the linear rate range identified in Fig. S3). Assays were conducted at 25 °C using 50 mm Bis tris propane, 50 mm NaCl, and 5 mm TCEP, pH 8.0, as buffer. Peptide concentrations of 5 times the relative *K_m_* for AtRAP2(2–15) were used for analysis (given in the graph titles) to ensure that AtPCO turnover was not limited by peptide availability with the exception of AtPCO4, which was analyzed at 1 mm AtRAP2(2–15) to avoid the substrate inhibition region of this isoform (Fig. 4). *Error bars* display S.E. (*n* = 3).

All of the AtPCOs demonstrated a clear dependence on O_2_ availability with respect to AtRAP2(2–15) oxidation rate. Michaelis–Menten kinetic plots (Fig. 5) allowed determination of *K*_*m*(O2)_^app^ values, which varied from 5.45% O_2_ for AtPCO5 to 17.3% O_2_ for AtPCO4 with AtPCOs 1, 2, and 3 generating constants of 15.7, 7.37, and 11.1% O_2_, respectively (Table 2). These quantities fall within the linear range of concentrations that can be measured by an optic fiber oxygen monitor, allowing headspace gas percentages to be converted into solution partial pressures. Consequently, corresponding *K*_*m*(O2)_^app^ values of 15.2, 6.57, 10.6, 16.5, and 4.83 kPa were determined for AtPCOs 1–5, respectively. The turnover numbers of AtPCOs 1 and 3 were higher than those obtained when measuring AtRAP2(2–15) dependence, indicating that their activity is submaximal at atmospheric O_2_ concentrations. This reflects their relatively high *K*_*m*(O2)_^app^ values, which are significantly greater than AtPCOs 2 and 5, isoforms that produced similar *k*_cat_ values in both experiments. In contrast, the turnover number of AtPCO4 was greater during AtRAP2(2–15) dependence assays as the influence of substrate inhibition is considered during the calculation of these kinetic parameters.

**Table 2:** **Steady-state kinetic parameters of AtPCOs 1–5 toward O_2_** The turnover numbers (*k*_cat_), Michaelis constants (*K_m_*), and related maximum velocities (*V*_max_) of AtPCOs 1–5 for O_2_ were calculated by analyzing single time points of AtPCO-catalyzed cysteine oxidation within the linear rate range established in Fig. S3 as a subject of O_2_ concentration using the Michaelis–Menten model of enzyme kinetics (Fig. 5). Gas headspace ratios (percent headspace) were converted into solution partial pressures of O_2_ (kPa solution) by directly monitoring concentration using an O_2_ probe.

AtPCO	*k*_cat_	*K_m_*	*K_m_*	*V*_max_
	*s*^−*1*^	%	*kPa solution*	μ*mol min*^−*1*^ *mg*^−*1*^
1	9.84 ± 0.45	15.7 ± 1.90	15.2 ± 1.84	16.8 ± 0.76
2	3.05 ± 0.09	7.37 ± 0.74	6.57 ± 0.66	5.56 ± 0.17
3	13.7 ± 0.40	11.1 ± 0.97	10.6 ± 0.92	24.6 ± 0.72
4	23.8 ± 0.90	17.3 ± 1.69	16.5 ± 1.61	48.6 ± 1.83
5	8.59 ± 0.25	5.45 ± 0.57	4.83 ± 0.51	17.5 ± 0.51

Significantly, the *K*_*m*(O2)_^app^ values determined are above or comparable with the O_2_ concentrations typically measured in vascular tissue and root structures (5–10% (23, 24)), indicating that the AtPCOs have the potential to act as physiological O_2_-sensing enzymes in plants.

### AtPCOs 1/2 and 4/5 show different AtERF-VII substrate preferences

Degradation of all five MetCys-initiating ERF-VII transcription factors from *Arabidopsis* (AtHRE1, AtHRE2, AtRAP2.2, AtRAP2.12, and AtRAP2.3) has been reported to be O_2_-dependent. Because of the sequence conservation at their N termini, it is likely that destabilization of all the AtERF-VIIs arises due to AtPCO-catalyzed N-terminal cysteine oxidation and subsequent N-end rule–mediated proteasomal degradation in normoxia. However, it is possible that different AtPCO isoforms preferentially target different AtERF-VII substrates; therefore, we sought to ascertain whether there was any substrate selectivity among the enzymes.

We first performed assays to confirm that 14-mer peptides corresponding to the methionine-excised N termini of AtHRE1, AtHRE2, and AtRAP2.3 (hereby referred to as AtHRE1(2–15), AtHRE2(2–15), and AtRAP2.3(2–15), respectively) were substrates for AtPCOs 1–5. Each peptide was incubated with AtPCOs 1–5 for 30 min at 25 °C before being analyzed by LC-MS similar to that described above. As expected, all AtPCO isoforms were capable of oxidizing each peptide (Figs. S4–S6), implicating AtPCO activity in the O_2_-dependent destabilization of all AtERF-VIIs *in vivo*.

**Figure 6.**
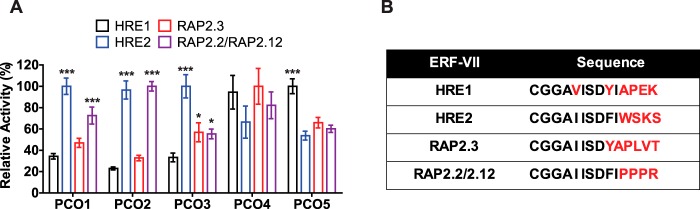
**AtERF-VII substrate preferences of AtPCOs 1–5.** *A*, the relative activities of AtPCOs 1–5 for each AtERF-VII substrate were determined by pooling the four peptides at a concentration equal to the *K_m_* for RAP2(2–15) and normalizing the rate of N-terminal cysteine oxidation to that of the best substrate. Specific activities are given in Table S1. Significant AtERF-VII preferences (relative to the poorest substrate) could be observed for most isoforms despite high conservation in the peptide sequences (*B*, where *black* indicates conservation with and *red* indicates deviation from RAP2.2/2.12). Statistical analysis was completed using a one-way analysis of variance, post hoc Dunnett test using the substrate that generated the lowest activity as the control reference, with * and *** denoting *p* ≤ 0.05 and *p* ≤ 0.001, respectively. *Error bars* display S.D. (*n* = 3).

To gain insight into the physiological function of each AtPCO, we next sought to investigate whether AtPCOs 1–5 have any preferences toward the AtERF-VII substrates. To this end, we used a competition assay in which the AtERF-VII peptides were pooled together, incubated with each AtPCO, and monitored for oxidation at regular time intervals. Equal concentrations of each peptide were used at a concentration equivalent to the respective *K_m_* for AtRAP2(2–15) for each AtPCO isoform. Rates of AtERF-VII oxidation were normalized to the optimal substrate for each enzyme such that substrate preferences could be easily compared between enzymes (Fig. 6*A*). Absolute rates of substrate oxidation are presented in Table S1.

We observed that each AtPCO isoform displayed different substrate preferences toward the AtERF-VII peptides despite high sequence conservation in the first nine amino acids of the peptide substrate (Fig. 6*B*). AtPCOs 1, 2, and 3 demonstrated selectivity for AtHRE2(2–15), whereas AtPCO2 and, to a lesser but significant extent, AtPCO1 also showed selectivity for AtRAP2(2–15). In contrast, AtPCO5 demonstrated selectivity toward AtHRE1(2–15), whereas AtPCO4 showed no significant preference for any AtERF-VII. The differences in substrate preference are likely due to the subtle differences in AtERF-VII sequence at the C terminus of the peptides (for example a Glu in position 14 of AtHRE1 and a Trp in position 12 of AtHRE2). Assuming these residues do not mechanistically contribute to N-terminal cysteine oxidation, this suggests that extensive interactions between the AtERF-VII N termini and the AtPCOs are important for substrate binding. Although these data were determined under isolated *in vitro* conditions, the results suggest that the AtPCO isoforms could have different biological roles.

## Discussion

ERF-VIIs enable transient survival in hypoxic conditions, such as those encountered during submergence, by up-regulating anaerobic response genes (1). ERF-VII stability is regulated in an O_2_-dependent manner by the PCOs, which use their cosubstrate to signal for ERF-VII degradation through the Cys/Arg branch of the N-end rule pathway (6–8, 13). This direct link among O_2_ availability, ERF-VII stability, and the hypoxic response has led to the suggestion that the PCOs act as O_2_ sensors, but their sensitivity to different substrates (including O_2_) has not been kinetically determined. Work described here defines the dependence of each PCO from *A. thaliana* on pH, AtRAP2.2/2.12, and O_2_ before comparing their activity with alternative AtERF-VII peptide substrates in an attempt to understand enzyme function.

While optimizing assay conditions, we found that AtPCO activity is susceptible to changes in pH with enzyme activity rapidly decreasing below pH 7.0 (Fig. 3*A*). This correlated with a side-group p*K_a_* of 6.78 for the N-terminal cysteine of RAP2(2–15) (which is low relative to the free amino acid), suggesting that this thiol needs to be deprotonated for substrate binding and/or turnover. Physiologically, PCO dependence on slightly basic conditions might act to enhance the plant hypoxic response as low-O_2_ conditions can reduce cytosolic pH by accumulating weak acids during anaerobic metabolism (25) and lowering ATP-driven proton pump activity at the plasma membrane, causing a buildup of H^+^ (26, 27). Accordingly, the cellular environment might influence PCO activity in addition to (co)substrate availability.

Kinetic constants were derived for AtPCOs 1–5 following steady-state analysis with respect to AtRAP2(2–15) and O_2_. The turnover numbers and Michaelis constants for each substrate varied considerably between different isoforms, but AtPCO4 retained the highest catalytic power in both instances. Its significantly greater *k*_cat_ appears to overcome the relatively weak *K_m_* for peptide and O_2_, allowing AtPCO4 to process large quantities of substrate even at low AtRAP2(2–15) and O_2_ concentrations. The expression of AtPCO4 is not up-regulated in hypoxia (in contrast to AtPCOs 1 and 2) (13), suggesting that it plays a constitutive role in RAP2.2/2.12 N-terminal cysteine oxidation. The high catalytic efficiency of AtPCO4 may provide plant cells with an underlying ability to target ERF-VIIs for removal both under normoxia and upon reoxygenation following hypoxia, offering continuous and general transcription factor maintenance. This is supported by the relatively broad substrate specificity of this isoform (Fig. 6), which hints that AtPCO4 can competently process all members of the AtERF-VII family.

AtPCO5 may also play a significant role in ERF-VII regulation *in vivo*. Although the turnover numbers calculated for this isoform are weaker than for AtPCO4, strong substrate association enables it to process large quantities of RAP2(2–15) at relatively low concentrations of peptide and O_2_, which may more accurately reflect physiological conditions.

It should be noted that the activity of both AtPCO4 and AtPCO5 was observed to decline at high AtRAP2(2–15) concentrations, indicating susceptibility to substrate inhibition. This could limit their ability to process AtERF-VII destabilization at high substrate levels. It is difficult to know if this apparent inhibition is a real property of AtPCO4/5 catalysis or the consequence of using a representative peptide as substrate. In either case, these negative effects were only detected above 1 mm, which might not be applicable *in planta*.

In contrast to AtPCOs 4 and 5, AtPCOs 1 and 2 (which are hypoxically induced (13)) demonstrated relatively low catalytic capability toward AtRAP2(2–15) and O_2_ but a clear substrate preference for AtHRE2 and AtRAP2.2/2.12. This raises the possibility that their up-regulation in hypoxia could help “turn off” the plant hypoxic response when O_2_ concentrations increase, particularly by targeting two of the most prominent anaerobic gene activators, AtRAP2.2/2.12, for degradation.

AtPCO3 showed preference for AtHRE2 but overall had relatively low kinetic capability (at least toward AtRAP2(2–15)) and is not hypoxically up-regulated (13). Although it is clear that it can contribute to AtERF-VII regulation, it must be considered for AtPCO3, as well as other isoforms, that there are over 200 MC-initiating proteins identified in *Arabidopsis* (28). It may be that there are alternative substrates for some or all of the AtPCOs that are more kinetically relevant than AtERF-VII oxidation.

We cannot rule out that additional interactions between the PCOs and full-length ERF-VIIs could influence their kinetic parameters. However, the peptides used represent N termini of transcription factors (often intrinsically disordered (29)) and as such are unlikely to be flanked by extensively structured regions. AtPCO kinetic parameters determined with the peptide substrates used in this study are therefore deemed likely to reflect their relative properties *in planta*; the use of peptide substrates is a strategy that has been widely used in the study of oxygen-sensing enzymes in animals (16, 30, 31).

A clear relationship between O_2_ concentration and AtPCO activity was observed for each isoform, highlighting their dependence on O_2_ for catalysis (Fig. 5 and Table 2). AtPCOs 2 and 5 have *K*_*m*(O2)_^app^ values of 7.37 and 5.45% (6.57 and 4.83 kPa), respectively, whereas AtPCOs 1 and 4 have higher *K*_*m*(O2)_^app^ values of 15.7 and 17.3% (15.2 and 16.5 kPa), respectively. AtPCO *K*_*m*(O2)_^app^ values are lower than those reported for mammalian O_2_-sensing enzymes, such as PHD2, which has a reported *K*_*m*(O2)_^app^ value of over 450 μm (equivalent to 40%, and at least twice the molar concentration calculated for the AtPCOs, as estimated by Henry's law) determined using the same technique (17). They also fall below standard atmospheric O_2_ concentrations in aqueous solution. However, all the isoforms showed a gradient of activity as oxygen concentrations depleted from normal levels.

Accurate measurement of O_2_ concentrations in plants is challenging and requires further technological optimization (32). Nevertheless, the lack of a circulatory system in plants dictates that O_2_ levels will be variable. Microprobes have been used to measure O_2_ in a number of different tissues (for a review, see Ref. 33), and steep gradients have been reported, ranging from near atmospheric levels in surface cells to mildly hypoxic (5–10% O_2_) in vascular tissues of the castor bean plant (24) and maize root meristems (23). O_2_ levels in seed embryos vary depending on coating and chlorophyll content (34) but can be near anoxic; *e.g.* sunflower seed embryos have O_2_ partial pressures of 0.08 kPa/<1 μm (35). O_2_ concentrations fall in potato tubers as they grow (to ∼5% (36)), and large fruit can also have O_2_ gradients from surface to core (*e.g.* 20–12 kPa in apples (37)). The *K*_*m*(O2)_^app^ values we report for the AtPCOs range from 5.45 to 17.3% (4.83–16.5 kPa; Table 2). Although these values were obtained under nonphysiological conditions, they nevertheless suggest that the activity of these enzymes is likely to be sensitive to physiologically relevant O_2_ gradients, thus enabling them to act as plant O_2_ sensors. It will be interesting to establish whether the expression of different PCO isoforms varies between different tissues depending on the O_2_ environment and enzyme sensitivity so that ERF-VII stability can be regulated appropriately.

Collectively, our results suggest that the AtPCOs are likely to be effective O_2_-sensing enzymes, which are capable of efficiently regulating the stability of ERF-VII transcription factors in response to hypoxia, potentially in a targeted manner. The relatively high activity of the constitutively expressed AtPCOs 4 and 5 could allow residual ERF-VIIs to be rapidly degraded under normoxic conditions, preventing the adverse consequences of anaerobic gene induction at regular oxygen concentrations. AtPCOs 1 and 2, whose expression is induced in hypoxia (13), could be responsible for terminating anaerobic processes when plants withdraw from hypoxia by targeting the primary activators RAP2.2/2.12 for degradation through the N-end rule pathway as soon as normal oxygen levels start to return. This would help alleviate the strain of prolonged fermentative metabolism, giving the organism the best chance of surviving transient periods of hypoxia, such as those experienced during flooding.

Our results reveal the potential for distinct roles for AtPCO isoforms. This raises the possibility that individual isoforms could be targeted for specific biological outcomes. Overexpression of AtRAP2.12 in *Arabidopsis* and stabilization of ERF-VII homologs in barley and rice have led to increased plant recovery following submergence (7, 38, 39). ERF-VIIs are also connected to tolerance of other forms of abiotic and biotic stress (12, 40). It is conceivable that a detailed understanding of PCO ERF-VII functionality, beginning with this kinetic study, could enable a rational approach to enhancing crop tolerance to a range of stresses through directed chemical or genetic inhibition methods.

## Experimental procedures

### Cloning and transformation of Atpco1–5 for heterologous expression in E. coli

The five *pco* genes from *A. thaliana* were amplified from initial constructs donated by Dr. Daan A. Weits and Professor Francesco Licausi using the Phusion polymerase system (New England Biolabs) and cloned into the NdeI and XhoI restriction sites of pET28a (Novagen) using ligation-independent methods (In-Fusion, Clontech). In-Fusion products were transformed into *E. coli* NEB5α competent cells (New England Biolabs) for insert validation by Sanger sequencing (Source Bioscience) before being transformed into *E. coli* BL21 (DE3) competent cells (New England Biolabs) for protein production.

### Heterologous expression of AtPCOs 1–5 in E. coli

*E. coli* transformants were grown in 600 ml of 2× Yeast extract Tryptone medium supplemented with 50 μm FeSO_4_ and 40 μg/ml kanamycin sulfate at 37 °C and 180 rpm until an *A*_600 nm_ was reached. Cultures were induced with 0.5 mm isopropyl 1-thio-β-d-galactopyranoside and grown overnight at 20 °C and 180 rpm before being harvested by centrifugation at 10,000 × *g* for 10 min.

### Purification of AtPCOs 1–5

Cell pellets were resuspended in 50 mm Tris and 400 mm NaCl, pH 7.5, supplemented with DNase and a Complete EDTA-free protease inhibitor mixture tablet and lysed by sonication. Cellular debris was removed by centrifugation at 48,000 × *g* for 30 min and filtration through a 0.45-μm membrane. The soluble supernatant was loaded onto an equilibrated HisTrap HP column (GE Healthcare) and washed with resuspension buffer containing increasing concentrations of imidazole until a definitive *A*_280 nm_ peak was collected. Contaminating proteins were removed using a HiLoad 26/600 Superdex 75 prep grade size-exclusion column (GE Healthcare) equilibrated with resuspension buffer. Protein purity was verified by SDS-PAGE, and protein concentrations were estimated using *A*_280 nm_ measurements obtained by a Cary UV-visible spectrometer.

### Metal quantification of AtPCOs 1–5

Enzyme iron concentrations were estimated using a bathophenanthroline disulfonic acid–based assay, which produces an absorbance reading at 535 nm in the presence of metal. Iron was extracted from protein samples by acid and heat denaturation, allowing absorbance comparison with a standard curve of known iron concentrations. Each sample contained a final concentration of 20% saturated ammonium acetate, 5 mm sodium metabisulfite, and 1 mm bathophenanthroline disulfonic acid as well as 0–250 μm Fe(II).

### Analyzing the activity of AtPCOs 1–5 with AtRAP2.2/2.12 at atmospheric O_2_

The activities of AtPCOs 1–5 were examined by incubating synthesized peptide (GL Biochem) corresponding to the first 14 amino acids of the methionine-excised N terminus of AtRAP2.2/2.12 (AtRAP2(2–15)), AtRAP2.3 (AtRAP3(2–15)), AtHRE1 (AtHRE1(2–15)), or AtHRE2 (AtHRE2(2–15)) with 0.1–1.0 μm enzyme in a benchtop thermocycler (Eppendorf) at 25 °C under aerobic conditions. 5 mm TCEP, 20 μm FeSO_4_, and 1 mm ascorbate were added to all samples to help maintain a reductive environment unless otherwise specified. 50 mm Bis tris propane and 50 mm NaCl were used to buffer most solutions with pH and peptide concentrations given in the text or figures. Time points were taken at regular intervals by quenching the reaction 1:10 with 1% formic acid for rate analysis. Oxidation was monitored by ultrahigh-performance LC (UPLC)-MS, and turnover was quantified by comparing the areas underneath the product and substrate ions extracted from the total ion current chromatogram. All figures and kinetic parameters were generated using Prism (GraphPad).

UPLC-MS measurements were obtained using an Acquity UPLC system coupled to a Xevo G2-S Q-ToF mass spectrometer (Waters) operated in positive electrospray mode. Instrument parameters, data acquisition, and data processing were controlled by MassLynx 4.1 with source conditions adjusted to maximize sensitivity and minimize fragmentation. Samples were injected onto a Chromolith Performance RP-18e 100 2-mm column (Merck) heated to 40 °C and eluted at 0.3 ml/min using a gradient of 95% deionized water supplement with 0.1% (v/v) formic acid to 95% acetonitrile.

### Determining the oxygen sensitivity of AtPCOs 1–5 with AtRAP2.2/2.12

The activities of AtPCOs 1–5 were examined at different O_2_ concentrations using peptide at 5 times the relevant *K_m_* for AtRAP2(2–15) unless otherwise specified. 100-μl aliquots of AtRAP2(2–15) were prepared as above, transferred into silicone-sealed MS vials, and equilibrated with different ratios of nitrogen and oxygen gas for 10 min using a mass flow controller (Brooks Instruments). Reactions were initiated by injecting 1 μl of enzyme using a gas-tight syringe (Hamilton) and allowed to proceed for 1 min (within the linear range of activity). Reactions were terminated by injecting 10 μl of 10% formic acid and analyzed as above, diluting the sample in 1% formic acid to avoid saturation of the detector if necessary. UPLC-MS analysis was carried out as described above.

The partial pressure of O_2_ in solution was estimated for each *K*_*m*(O2)_^app^ using an Oxylite probe (Oxford Optronix), which measures concentrations in the physiological range. Buffer samples equilibrated with the relevant gas ratios were prepared as above and tested for O_2_ concentration by inserting the probe through a needle. Multiple readings were taken to ensure accuracy, giving a standard error of 0.37 kPa for PCO1, 0.07 kPa for PCO2, 0.33 kPa for PCO3, 0.23 kPa for PCO4, and 0.03 kPa for PCO5.

### Comparing the activity of AtPCOs 1–5 with different ERF-VII substrates at atmospheric oxygen

The activities of AtPCOs 1–5 with AtRAP2.2/2.12 (AtRAP2(2-15)), AtRAP2.3 (AtRAP3(2–15)), AtHRE1 (AtHRE1(2–15)), and AtHRE2 (AtHRE2(2–15)) were compared using a competition assay where all substrates were pooled at a concentration equivalent to the relevant *K_m_* for AtRAP2(2–15) and analyzed for turnover rate at 25 °C as described above under aerobic conditions.

### Determining the thiol pK_a_ of AtRAP2.2/2.12 using NMR

A selection of 10 buffer solutions of NaH_2_PO_4_ (50 mm) in D_2_O were prepared at a set range of pH values ranging from 5.60 to 7.85 with 0.25 increments (pH* 5.60, 5.85, 6.10, 6.35, 6.60, 6.85, 7.10, 7.35, 7.60, and 7.85). The peptide (1.03 mg) was dissolved in D_2_O (160 μl). A stock solution of 1,4-dioxane (12.9 μl) was prepared by dissolving in D_2_O (1.5 ml) and used as a reference. A total of 10 samples were prepared at the different pH* values by mixing buffer (152 μl), peptide solution (8 μl) and 1,4-dioxane stock (1 μl). Samples were subsequently analyzed by ^1^H NMR at 298 K.

NMR experiments were carried out on a Bruker Avance III 700 MHz equipped with a TCI inverse cryoprobe with sample temperature regulated at 298 K. Data were analyzed using Bruker Topspin 3.5. Processing of spectra was done with a Lorentzian line broadening of 0.3 Hz. Chemical shifts (δ) are given as ppm relative to 1,4-dioxane (δ_H_ 3.68 ppm for ^1^H NMR). The p*K_a_* value for H_2_O was established using the formula derived previously (41).

## Author contributions

M. D. W. formal analysis; M. D. W. and E. F. supervision; M. D. W. and E. F. validation; M. D. W., J. J. A. G. K., S. E., L. J. T. K., and E. F. investigation; M. D. W. and E. F. visualization; M. D. W., J. J. A. G. K., S. E., L. J. T. K., and E. F. methodology; M. D. W. and E. F. writing-original draft; M. D. W. and E. F. project administration; M. D. W., J. J. A. G. K., S. E., L. J. T. K., and E. F. writing-review and editing; E. F. conceptualization; E. F. funding acquisition.

## Supplementary Material

Supporting Information

## Footnotes

5 The abbreviations used are:

ERF-VII: group VII ethylene response factor
PCO: plant cysteine oxidase
At: *A. thaliana*
kPa: kilopascal(s)
HIF: hypoxia-inducible transcription factor
PHD: prolyl hydroxylase enzyme
Bis tris propane: 1,3-bis[tris(hydroxymethyl)methylamino]propane
TCEP: tris(2-carboxyethyl)phosphine
UPLC: ultrahigh-performance LC.
