# Supplementary material for: The plant cysteine oxidases from Arabidopsis thaliana are kinetically tailored to act as oxygen sensors
Source: J Biol Chem. 2018 May 30;293(30):11786–95. doi: 10.1074/jbc.RA118.003496 (PMC6066304; doi:10.1074/jbc.RA118.003496)
Supplement: Supporting Information [file supp_293_30_11786__index.html]

The Plant Cysteine Oxidases from Arabidopsis thaliana are kinetically tailored to act as oxygen sensors — Plant Cysteine Oxidase oxygen kinetics — The plant cysteine oxidases from Arabidopsis thaliana are kinetically tailored to act as oxygen sensors — Plant cysteine oxidase oxygen kinetics — Supporting Information 

# The plant cysteine oxidases from *Arabidopsis thaliana* are kinetically tailored to act as oxygen sensors

## Supporting Information

- White et al JBC\_2018\_003496\_Supplementary\_Information - Supplementary Information file
