# Supplementary material for: The plant cysteine oxidases from Arabidopsis thaliana are kinetically tailored to act as oxygen sensors
Source: J Biol Chem. 2018 May 30;293(30):11786–95. doi: 10.1074/jbc.RA118.003496 (PMC6066304; doi:10.1074/jbc.RA118.003496)
Supplement: Supporting Information [file supp_RA118.003496_137492_1_supp_144144_p9hhzy.pdf]

The Plant Cysteine Oxidases from *Arabidopsis thaliana* are kinetically tailored to act as oxygen sensors

Mark D. White*, Jos J. A. G. Kamps, Samuel East, Leah J. Taylor Kearney, and Emily Flashman*

Address: Chemistry Research Laboratory, University of Oxford, 12 Mansfield Road, Oxford, OX1 3TA, United Kingdom

Running title: Plant Cysteine Oxidase oxygen kinetics

Supplementary Information

Supplementary Tables

PCO	HRE1 (μmol min^{-1} mg^{-1})	HRE2 (μmol min^{-1} mg^{-1})	RAP2.3 (μmol min^{-1} mg^{-1})	RAP2.2/2.12 (μmol min^{-1} mg^{-1})
1	0.95 ± 0.04	2.77 ± 0.12	1.30 ± 0.07	2.01 ± 0.13
2	0.33 ± 0.01	1.40 ± 0.07	0.47 ± 0.02	1.45 ± 0.04
3	0.63 ± 0.04	1.89 ± 0.12	1.07 ± 0.10	1.04 ± 0.05
4	3.52 ± 0.34	2.48 ± 0.32	3.73 ± 0.36	3.07 ± 0.27
5	4.20 ± 0.17	2.25 ± 0.10	2.77 ± 0.12	2.52 ± 0.08

Table S1 – The specific activities of AtPCO 1 to 5 with different AtERF-VII substrates: The specific activities of each AtPCO isoform with different AtERF-VII peptides, calculated from a competition assay where all substrates were pooled at a concentration equal to the relative K_m for $RAP2_{2-15}$. Values were normalized to the substrate that generated the greatest activity for individual AtPCOs to produce Figure 6A.

Supplementary Figures

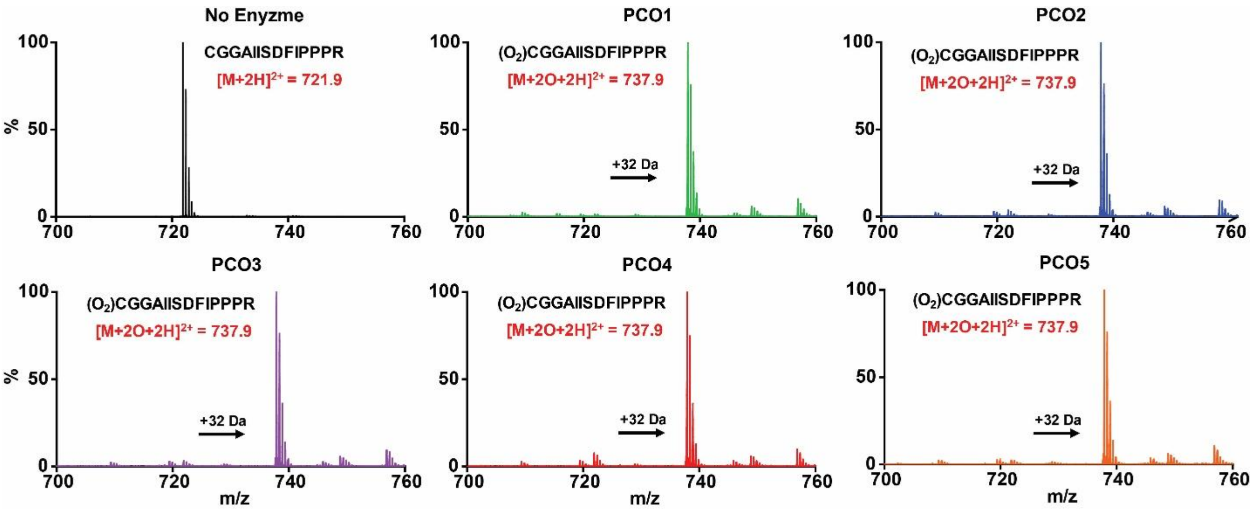

Figure S1 - AtPCOs 1 to 5 catalyze dioxygenation of AtRAP2$_{2\text{-}15}$: LC-MS spectra demonstrating the AtRAP2$_{2\text{-}15}$ mass species detected following 30 minute incubation with and without AtPCO 1 to 5 under aerobic conditions at 25 ^{o}C. An enzyme-dependent mass increase of +32 Da, consistent with Cys-sulfinic acid formation (1), was observed in all cases. 50 mM HEPES, 50 mM NaCl and 1 mM TCEP pH 7.5 was used as buffer.

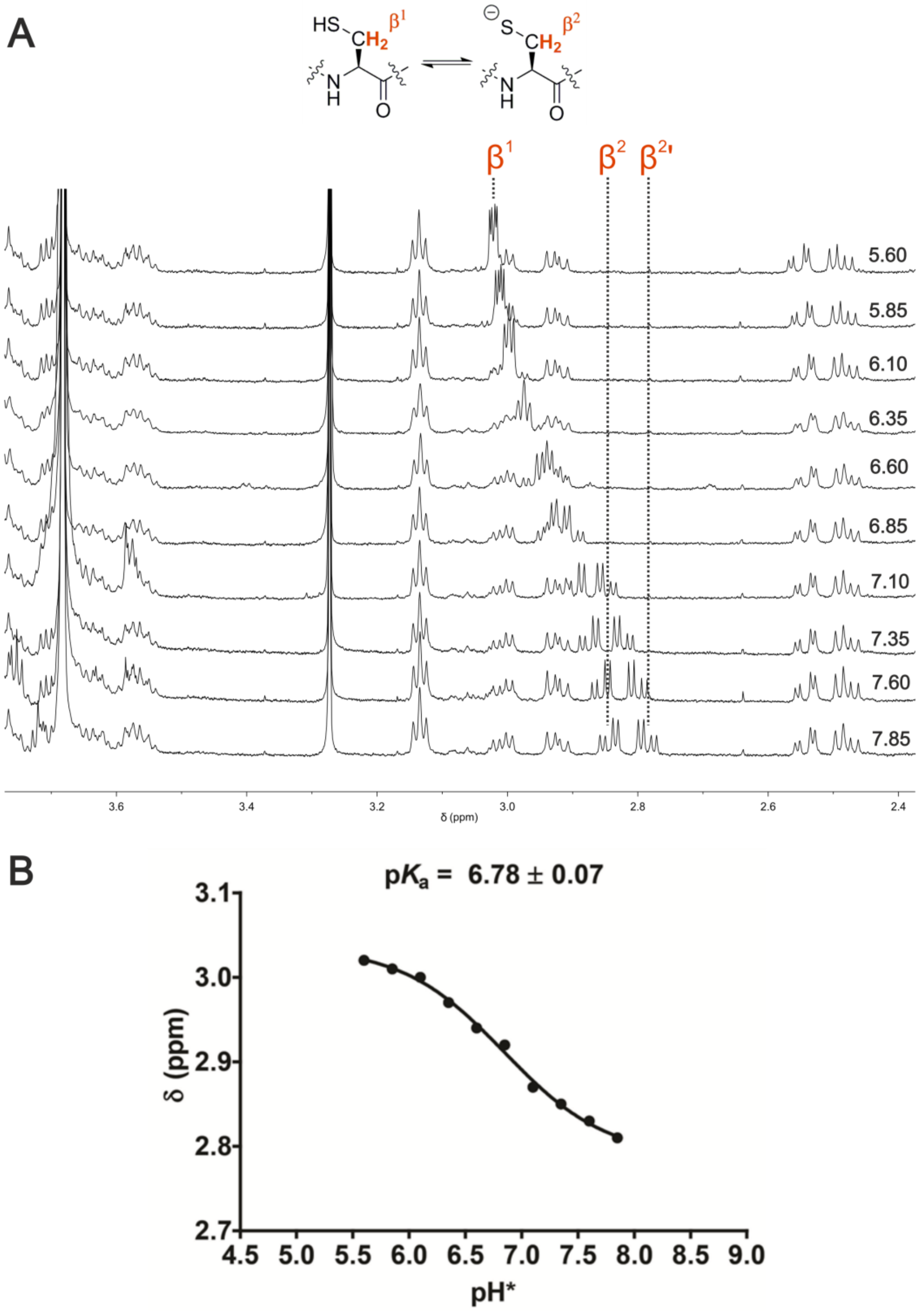

Figure S2 - ^{1}H NMR analysis of AtRAP2$_{2\text{-}15}$: A Stacked ^{1}H NMR spectra of AtRAP2$_{2\text{-}15}$ peptide (223 μM) in D_2O at different pH* (5.60 to 7.85), using 50 mM NaH_2PO_4 as buffer. Chemical shifts following the cysteine β-protons (consistent with deprotonation of the N-terminal thiol group, in the case of the deprotonated diastereotopic β-protons, pH 6.8 and above, the average of the chemical shift is used) were observed at higher pH* which allowed the pK_a of the cysteine side group in H_2O to be estimated (Figure 3B) using the equation, $pK^H = 0.929pK^{H*} + 0.42$, given in (2); **B** plotted pH versus chemical shift δ (ppm).

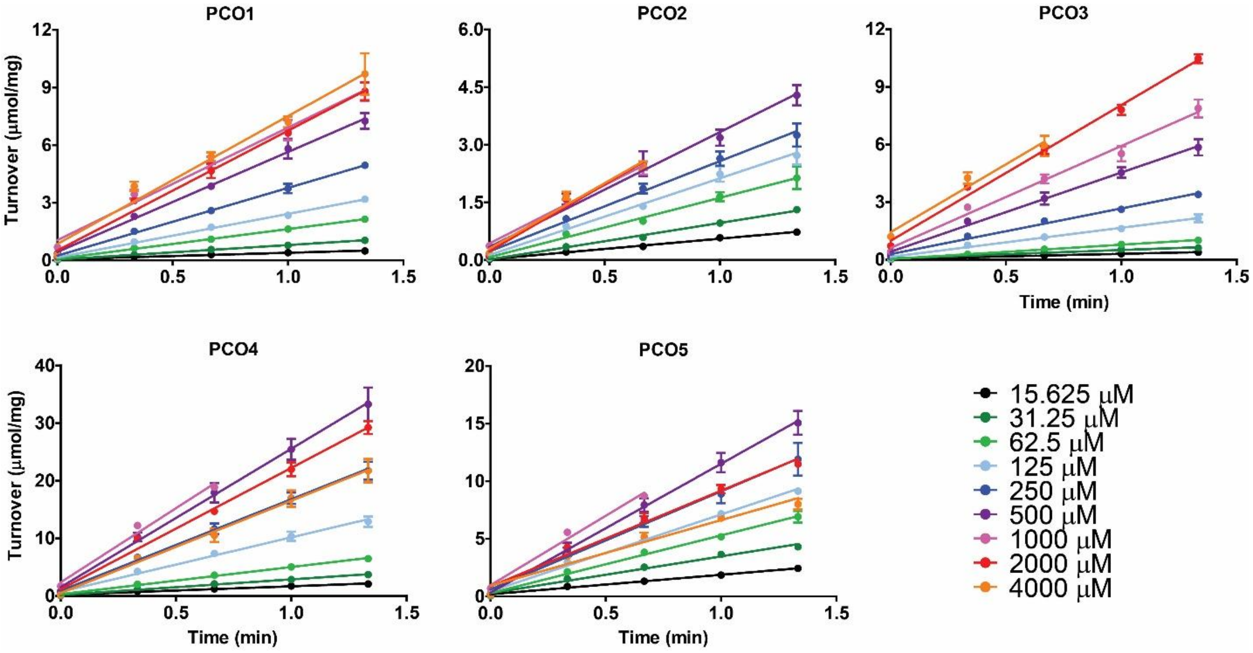

Figure S3 – Initial rate data for AtPCOs 1 to 5 with different concentrations of AtRAP2$_{2\text{-}15}$: Rate profiles for AtPCOs 1 to 5 with different concentrations of AtRAP2$_{2\text{-}15}$, indexed in the figure. Assays were conducted under aerobic conditions at 25 ^{o}C, using 50 mM bis-tris propane, 50 mM NaCl and 5 mM TCEP pH 8.0 as buffer and analyzed by LC-MS. This data was used to produce the Michaelis-Menten kinetic plots shown in Figure 4. Standard error is shown (n=3).

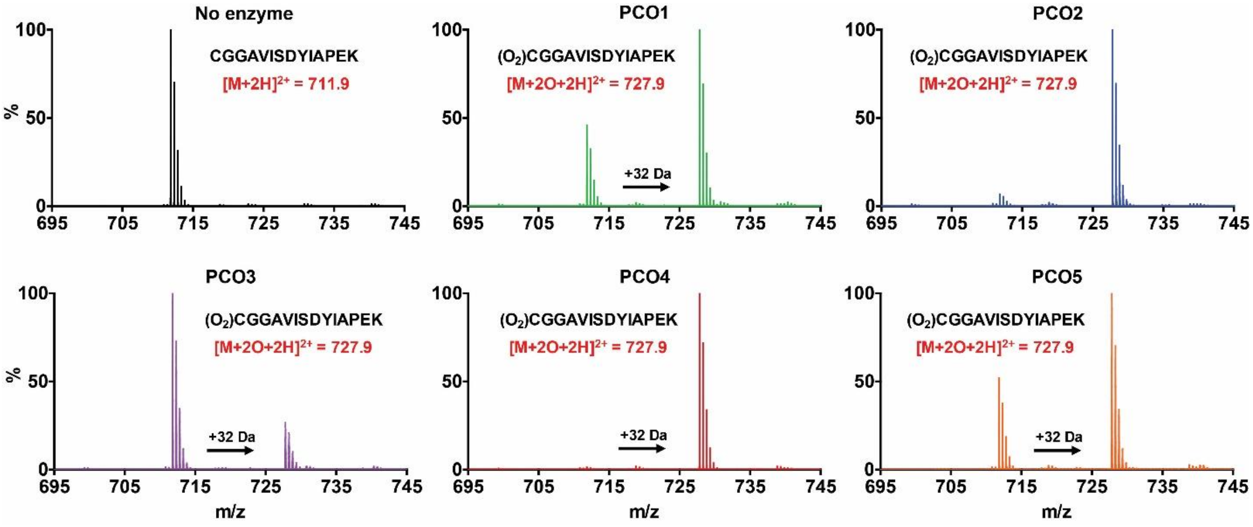

Figure S4 - AtPCOs 1 to 5 catalyze dioxygenation of AtHRE1$_{2\text{-}15}$: LC-MS spectra demonstrating the AtHRE1$_{2\text{-}15}$ mass species detected following 30 minute incubation with and without AtPCO 1 to 5 under aerobic conditions at 25 ^{o}C. An enzyme-dependent mass increase of 32 Da, consistent with Cys-sulfinic acid formation (1), was observed in all cases. 50 mM HEPES, 50 mM NaCl and 1 mM TCEP pH 7.5 was used as buffer.

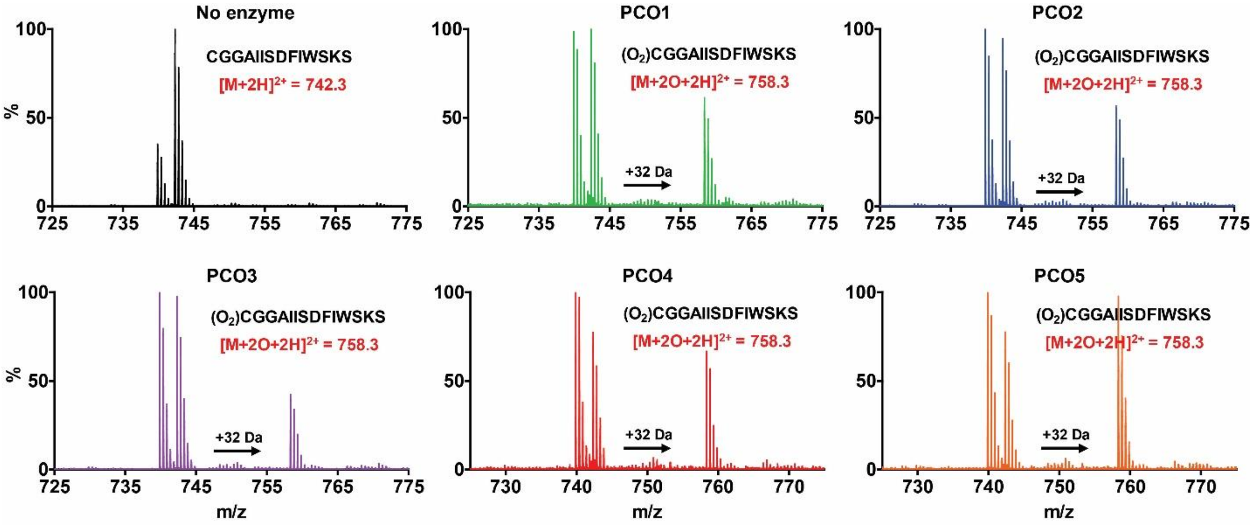

Figure S5 - AtPCOs 1 to 5 catalyze dioxygenation of AtHRE2$_{2\text{-}15}$: LC-MS spectra demonstrating the RAP2$_{2\text{-}15}$ mass species detected following 30 minute incubation with and without AtPCO 1 to 5 under aerobic conditions at 25 ^{o}C. An enzyme-dependent mass increase of 32 Da, consistent with Cys-sulfinic acid formation (1), was observed in all cases. A contaminating peak of 739.9 Da was observed in all spectra, which could not be identified. 50 mM HEPES, 50 mM NaCl and 1 mM TCEP pH 7.5 was used as buffer.

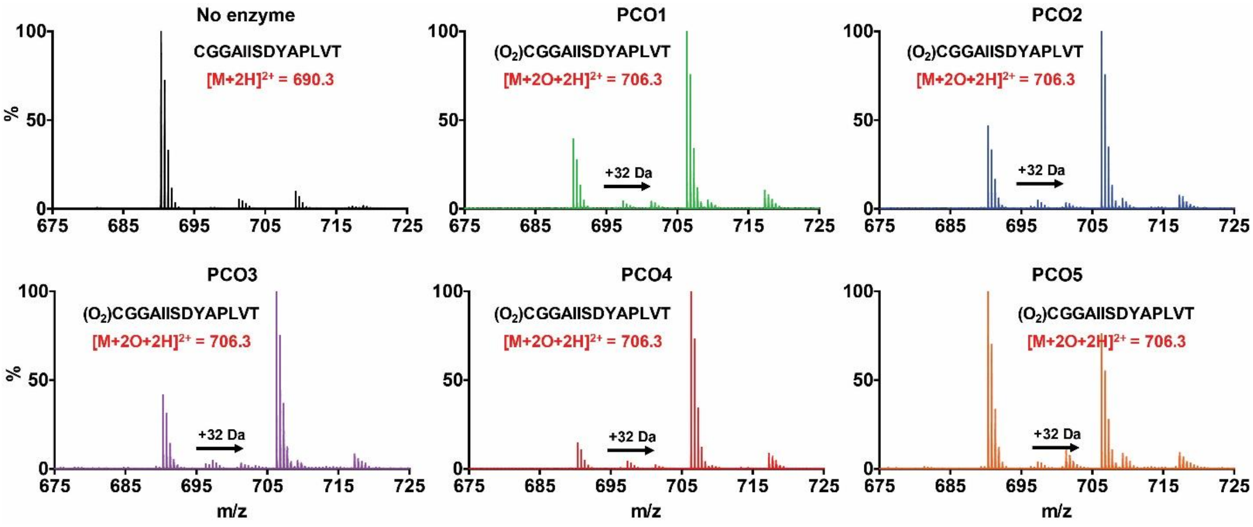

Figure S6 - AtPCOs 1 to 5 catalyze dioxygenation of AtRAP2.3$_{2\text{-}15}$: LC-MS spectra demonstrating the AtRAP2.3$_{2\text{-}15}$ mass species detected following 30 minute incubation with and without AtPCO 1 to 5 under aerobic conditions at 25 ^{o}C. An enzyme-dependent mass increase of 32 Da, consistent with Cys-sulfinic acid formation (1), was observed in all cases. 50 mM HEPES, 50 mM NaCl and 1 mM TCEP pH 7.5 was used as buffer.

References

1. White, M. D., Klecker, M., Hopkinson, R. J., Weits, D. A., Mueller, C., Naumann, C., O'Neill, R., Wickens, J., Yang, J., Brooks-Bartlett, J. C., Garman, E. F., Grossmann, T. N., Dissmeyer, N., and Flashman, E. (2017) Plant cysteine oxidases are dioxygenases that directly enable arginyl transferase-catalysed arginylation of n-end rule targets *Nature communications* **8**, 14690

2. Krężel, A., and Bal, W. (2004) A formula for correlating pKa values determined in D_2O and H_2O *J Inorg Biochem* **98**, 161-166
